# Patterns of cytokine release and association with new onset of post-cardiac surgery atrial fibrillation

**DOI:** 10.3389/fsurg.2023.1205396

**Published:** 2023-05-30

**Authors:** Rahul Kota, Marco Gemelli, Arnaldo Dimagli, Saadeh Suleiman, Marco Moscarelli, Tim Dong, Gianni D. Angelini, Daniel P. Fudulu

**Affiliations:** ^1^ Bristol Medical School, University of Bristol, Bristol, United Kingdom; ^2^ Department of Cardiac Surgery, Bristol Heart Institute, Bristol, United Kingdom

**Keywords:** cardiac surgery, cytokines, post-operative atrial fibrillation, aortic valve replacement, coronary artery bypass grafting

## Abstract

**Introduction:**

Postoperative Atrial Fibrillation (POAF) is a common complication of cardiac surgery, associated with increased mortality, stroke risk, cardiac failure and prolonged hospital stay. Our study aimed to assess the patterns of release of systemic cytokines in patients with and without POAF.

**Methods:**

A post-hoc analysis of the Remote Ischemic Preconditioning (RIPC) trial, including 121 patients (93 males and 28 females, mean age of 68 years old) who underwent isolated coronary artery bypass grafting (CABG) and aortic valve replacement (AVR). Mixed-effect models were used to analyze patterns of release of cytokines in POAF and non-AF patients. A logistic regression model was used to assess the effect of peak cytokine concentration (6 h after the aortic cross-clamp release) alongside other clinical predictors on the development of POAF.

**Results:**

We found no significant difference in the patterns of release of IL-6 (*p* = 0.52), IL-10 (*p* = 0.39), IL-8 (*p* = 0.20) and TNF-α (*p* = 0.55) between POAF and non-AF patients. Also, we found no significant predictive value in peak concentrations of IL-6 (*p* = 0.2), IL-8 (*p* = >0.9), IL-10 (*p* = >0.9) and Tumour Necrosis Factor Alpha (TNF-α)(*p* = 0.6), however age and aortic cross-clamp time were significant predictors of POAF development across all models.

**Conclusions:**

Our study suggests no significant association exists between cytokine release patterns and the development of POAF. Age and Aortic Cross-clamp time were found to be significant predictors of POAF.

## Introduction

Postoperative atrial fibrillation (POAF) is a common complication of cardiac surgery, with the incidence rate being up to 60%, depending on the type of surgery (1). It is associated with an increased risk of early and long-term mortality, early and long-term stroke, renal impairment, cardiac failure and haemodynamic instability (2, 3). It is known that surgery with the use of cardiopulmonary bypass (CPB) elicits a systemic inflammatory response syndrome (SIRS) and the release of systemic cytokines that have been suggested to be associated with the development of POAF (4–7). Furthermore, it is thought that inflammation can alter the electrophysiology and structure of the heart leading to increased vulnerability to atrial fibrillation (8) (AF).

The aim of this study was to evaluate the hypothesis that systemic cytokines, particularly IL-6, IL-8, IL-10 and TNF-α, would have significantly different patterns of release in patients with and without POAF undergoing coronary artery bypass grafting (CABG) or aortic valve replacement (AVR) surgery. To test this hypothesis, we undertook a post-hoc analysis of the remote ischemic preconditioning (RIPC) trial database.

## Materials and methods

The RIPC trial was approved by the London-Harrow Research Ethics Committee (reference number REC number 12/LO/1361) and was registered to the International Standard Randomized Controlled Trial Number (ISRCTN) registry with the ID 33084113 (doi: 10.1186/ISRCTN33084113). The RIPC trial aimed to assess the effect of remote ischaemic preconditioning in 124 patients undergoing isolated CABG and AVR on cardiac injury, metabolic stress, and inflammatory response (9) between February 2013 and April 2015. The RIPC intervention comprised four 5-min cycles of upper limb ischaemia, induced by a blood pressure cuff inflated to 200 mmHg, followed by a 5 min period of reperfusion by deflating the cuff. The expression of relevant cytokines was assessed using the MILLIPLEX® MAP Human High Sensitivity T Cell Magnetic Bead Panel as per the RIPC trial. Cytokines were measured at baseline (before the operation) and 6, 12, 24, 48 and 72 h after the aortic cross-clamp release. For the cytokine analysis, we have used GraphPad Prism version 8.4.3 GraphPad Software, La Jolla California USA, www.graphpad.com. Because some cytokine measurements were missing per time-point, we performed the analysis by fitting a mixed-effects model to assess changes in nucleotide metabolism between AF and non-AF patients. We tested for data outlier cytokine measurements using a ROUT method (10) and removed from the analysis: 1 outlier in the IL-6 data, 21 in the IL-10 data, 1 outlier in the IL-8 data and 9 outliers in the TNF-α data. For the analysis of baseline characteristics, we used R version 1.4.1717, gtsummary. Categorical variables were summarised as counts and percentages and compared by the chi-square test, Continuous variables were summarised as mean and standard deviation (SD) or median and interquartile range, as appropriate per their distribution tested with Shapiro-Wilks test, and compared using t-test or rank-sum test. Three patients in the original trial had preoperative AF and were excluded from the analyses. We used logistic regression models to assess the effect of each cytokine at 6 h post-reperfusion (the returning of blood to the heart) along with age and cross-clamp time. We have chosen to include in the model the 6-hour post-reperfusion time point because this is when the cytokine concentrations peaked. We have performed subgroup analysis for patients undergoing AVR or CABG to assess wether cytokine have a procedure-specific effect.

Of note, in the RIPC trial, the remote ischaemic preconditioning intervention was no effect compared to the sham on the outcomes of interest, including cytokine patterns. Therefore, we have included in the post-hoc analysis both arms of the study (9) (sham and intervention) and performed the analysis. However, we must acknowledge that this post-hoc trial analysis should be viewed as hypothesis-generating only (11, 12). The AF outcome was recorded during hospitalization and was not followed- up on discharge. None of the patients (CABG or AVR) had a posterior pericardiotomy performed.

## Results

### Baseline characteristics of POAF and No-AF cohorts

Data was analyzed for 121 patients (77% male, 23% female, mean age 68 years old). Sixty-four patients (53%) underwent CABG, while 57 (47%) underwent AVR. Of these, 32% experienced POAF (*N* = 39, 26 males, mean age of 74). Patient characteristics and risk factors were broadly balanced between groups, with few statistically significant differences between the POAF and No-AF cohorts (Table 1). The POAF cohort patients were significantly older (74 vs. 65) (*p* = <0.001), more likely to be in NYHA Class III (21% vs. 17%) (*p* = 0.003), had a longer time on CPB (95 min vs. 82 min) (*p* = 0.003), and aorta cross clamped time (63 min vs. 48 min) (*p* = <0.001).

**Table 1:** Baseline characteristics of AF and non-AF patients.

Characteristic	Overall, *N* = 121^1^	No AF, *N* = 82^1^	AF, *N* = 39^1^	*p*-value^2^
Procedure				**<0**.**001**
CABG	64 (53%)	54 (66%)	10 (26%)	
AVR	57 (47%)	28 (34%)	29 (74%)	
Age(years)	68 (61, 75)	65 (58, 71)	74 (68, 76)	**<0**.**001**
Sex				0.067
Male	93 (77%)	67 (82%)	26 (67%)	
Females	28 (23%)	15 (18%)	13 (33%)	
Body mass index (kg/m2)	27.5 (25.1, 32.5)	27.4 (24.9, 32.3)	27.5 (25.4, 32.7)	>0.9
CCS class				0.10
0	31 (26%)	15 (18%)	16 (41%)	
1	27 (22%)	19 (23%)	8 (21%)	
2	52 (43%)	39 (48%)	13 (33%)	
3	10 (8.3%)	8 (9.8%)	2 (5.1%)	
4	1 (0.8%)	1 (1.2%)	0 (0%)	
NYHA class				**0**.**003**
1	28 (23%)	26 (32%)	2 (5.1%)	
2	70 (58%)	41 (50%)	29 (74%)	
3	22 (18%)	14 (17%)	8 (21%)	
4	1 (0.8%)	1 (1.2%)	0 (0%)	
LV function				0.4
Good (EF > 50%)	100 (83%)	66 (80%)	34 (87%)	
Poor (<50% and >30%)	21 (17%)	16 (20%)	5 (13%)	
Creatinine (mg/dl)	81 (72, 98)	81 (73, 99)	83 (70, 94)	0.7
Previous CVA/TIA	11 (9.1%)	8 (9.8%)	3 (7.7%)	>0.9
Smoking status				0.8
Smoking	13 (11%)	10 (12%)	3 (7.7%)	
Ex-smoking	56 (46%)	37 (45%)	19 (49%)	
Peripheral vascular disease	4 (3.3%)	2 (2.4%)	2 (5.1%)	0.6
Pulmonary disease	25 (21%)	15 (18%)	10 (26%)	0.4
Neurological dysfunction	1 (0.8%)	1 (1.2%)	0 (0%)	>0.9
Previous MI	18 (15%)	15 (18%)	3 (7.7%)	0.13
Hypertension	97 (80%)	66 (80%)	31 (79%)	0.9
Hypercholesterolaemia	91 (75%)	66 (80%)	25 (64%)	0.051
Diabetes				>0.9
NIDDM	26 (21%)	17 (21%)	9 (23%)	
IDDM	5 (4.1%)	4 (4.9%)	1 (2.6%)	
Pre-op beta-blocker	67 (55%)	47 (57%)	20 (51%)	0.5
Pre-op statin	88 (73%)	63 (77%)	25 (64%)	0.14
Cardiopulmonary bypass time (min)	84 (74, 101)	82 (71, 94)	95 (80, 113)	**0**.**003**
Aortic cross-clamp time (min)	51 (39, 65)	48 (37, 56)	63 (50, 81)	**<0**.**001**

AVR, aortic valve replacement; CSS, Canadian cardiovascular society; CABG, coronary artery bypass grafting; IDDM, insulin-dependent diabetes mellitus; LV, left ventricular; NYHA, New York heart association; NIDDM, non-insulin-dependent diabetes mellitus; TIA, transient ischaemic attack; CVA, cerebrovascular accident.

^1^ *n* (%); Median (IQR).

^2^ Pearson's Chi-squared test; Wilcoxon rank sum test; Fisher's exact test.

### Effect of postoperative AF on mortality, stroke and length of hospital stay

The outcomes for the POAF cohort and no-AF cohort are presented in Table 2. There was no statistically significant difference in the occurrence of cerebrovascular accident (CVA) or transient ischemic attack (TIA) between POAF and non-AF patients (7.7% vs. 9.8%, (*p* = >0.9). However, there was a statistically significant increase in hospital stay for POAF patients compared to no-AF patients (8 days vs. 6 days, *(p* = 0.002).

**Table 2:** Outcomes of AF and non-AF cohorts.

Characteristic	Overall, *N* = 121^1^	No AF, *N* = 82^1^	AF, *N* = 39^1^	*p*-value^2^
Mortality	0 (0%)	0 (0%)	0 (0%)	
CVA or TIA	11 (9.1%)	8 (9.8%)	3 (7.7%)	>0.9
Hospital stays (days)	7 (6, 8)	6 (5, 8)	8 (6, 10)	0.002

CVA, cerebrovascular accident; TIA, transient ischemic attack.

^1^ *n* (%); Median (IQR).

^2^ Fisher's exact test; Wilcoxon rank sum test.

### Analysis of cytokine profiles

**Figure 1:**
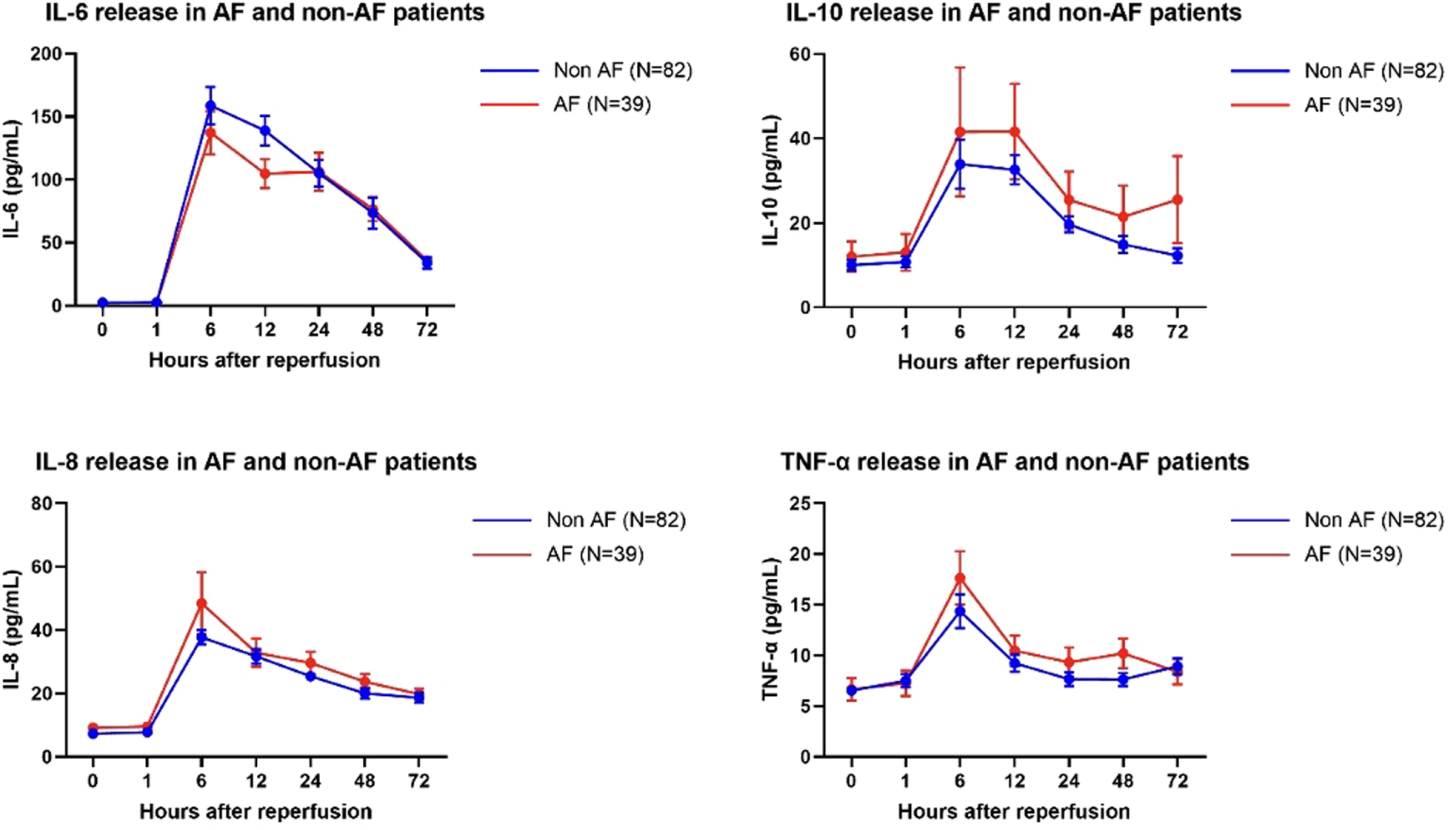
IL-6, IL-10, IL-8 and TNFα concentration a baseline and at 1, 6, 12, 24, 48 and 72 h postreperfusion in AF and non-AF patients. Data are the mean ± SEM, data were analyzed using a mixed model.

After fitting a mixed-effects model, we found no significant difference in the patterns of release of IL-6 (*p* = 0.52), IL-10 (*p* = 0.39), IL-8 (*p* = 0.20) and TNF-α (*p* = 0.55) between POAF and non-AF patients (Figure 1).

### Effect of cytokine concentration in prediction for post-op AF

Using a logistic regression model, we tested whether the peak concentration of each of the cytokines analyzed is a predictor for POAF alongside age and cross-clamp times (Table 3). We found no significant predictive value in peak concentrations of IL-6 (*p* = 0.2), IL-8 (*p* = >0.9), IL-10 (*p* = >0.9) and Tumour Necrosis Factor Alpha (TNF-α)(*p* = 0.6), however age and aortic cross-clamp time were significant predictors of POAF across all 4 models. We have also performed subgroup analyses of patients undergoing aortic valve replacement or coronary artery bypass grafting and found no sinficant effect of peak cytokines on development of atrial fibrillation (Table 1 and Supplementary Table 2).

**Table 3:** The effect of IL-6, IL-0, IL-8 and TNFα in logistic regression models for prediction of POAF.

*Predictors*	IL-6 model			IL-10 model			IL-8 model			TNFα-model		
	*Odds Ratios*	*CI*	*p*	*Odds Ratios*	*CI*	*p*	*Odds Ratios*	*CI*	*p*	*Odds Ratios*	*CI*	*p*
Age (years)	1.09	1.04–1.15	**0.001**	1.09	1.04–1.15	**0.001**	1.09	1.04–1.15	**0.001**	1.09	1.04–1.14	**0.001**
Aortic cross clamp time (min)	1.04	1.01–1.06	**0.003**	1.04	1.02–1.06	**0.001**	1.04	1.02–1.06	**0.001**	1.04	1.02–1.06	**0.001**
IL-6 (pg/ml)	1.00	0.99–1.00	0.156									
IL-10 (pg/ml)				1.00	0.99–1.01	0.911						
IL-8 (pg/ml)							1.00	0.99–1.01	0.911			
TNFα (pg/ml)										1.01	0.98–1.03	0.616
Observations	118			119			119			119		
R^2^ Tjur	0.256			0.244			0.244			0.245		

Bold values indices are statistically significant of *p*-value.

## Discussion

Cardiac surgery with the use of CPB stimulates the release of proinflammatory and anti-inflammatory cytokines as part of the systemic inflammatory response to surgery (SIRS) (13–15). SIRS after cardiac surgery is multifactorial: contact activation of plasma proteins as blood encounters artificial surfaces of the bypass circuit (16), ischaemic reperfusion injury to the brain (17), heart (18), lungs (19) and other organs as a by-product of aortic cross-clamping and endotoxemia (13). It has been shown that pro-inflammatory cytokines play a crucial role in fuelling the inflammatory process, with TNF-α concentrations peaking early after cardiac surgery and IL-6 and IL-8 concentrations peaking later (20, 21).

It has been suggested that a significant, systemic increase in cytokine concentrations in the blood may contribute to an increased risk of POAF. In particular, increased levels of IL-6 and TNF-α post-operatively have been reported to be associated with POAF (22–25). Furthermore, Maesen et al. (26) suggested that corticosteroids reduce the incidence of POAF due to their inhibition of cytokine release. It is unclear to what extent the systemic cytokine response correlates to inflammation at an atrial tissue since we could not perform a histological analysis in these atrial samples. However, it has been shown that atrial fibrillation is indeed associated with local, atrial tissue inflammation that promotes oxidative stress and electrical instability.

The main finding of our study was that in patients undergoing isolated CABG or AVR, there were no significant differences in the patterns of release of cytokines IL-6, IL-10, IL-8 and TNF-α between those that developed postoperative atrial fibrillation and those who did not. Fruthemore, we found no effect of cytokines on development of POAF in patients undergoing atrial fibrillation.

Our results mirror the report from Wu et al. (7) who in a study of 113 CABG patients found that IL-6, IL-8 and IL-10 all had similar patterns of release in patients with POAF and also non-AF patients. Our findings are also similar to Ishida et al. (27), who provided evidence that TNF-α and IL-8 are released in similar patterns for patients who developed POAF and those who did not. In contrast to the work by Ishida et al, we found no difference in IL-6 release patterns between patients who developed POAF and non-AF patients. Furthermore, IL-6 was not a significant predictor for the development of POAF (4–7).

Our study also concluded that older age was an independent risk factor for the development of POAF*,* similar to the report of Mathew et al. (28), which showed a 10-year increase in age increases AF odds by 75%*.* Additionally, Todorov et al. (29) determined that there was an odds ratio of 1.448 per decade increase in age in the development of AF. Turkkolu et al. (30) found older age to be a statistically significant predictor of POAF following cardiac surgery.

A longer aortic cross-clamp time was found to be a statistically significant factor in the development of POAF (Table 3)*.* Our data supports a Hashemzadeh et al. (31) study which also concluded that a longer aortic cross-clamp time is associated with POAF development (*p* = 0.040). Further data collected by Qureshi et al. (32) also agreed with our conclusion of an increased aortic cross-clamp time and proposed aortic cross-clamping time should be kept under 60 min.

As well as a longer aortic cross-clamp time, we found that a longer time spent on CPB was a statistically significant factor in the development of POAF (*p* = 0.003). Again, this aligns with Hashemzadeh et al. (31) whose study found statistical significance in increased CPB time correlating to POAF development.

Finally, our study found the POAF cohort spent significantly longer in hospital (2 days more); similar to the report of Park et al. (33), which concluded POAF patients had an average postoperative stay 2.4 days longer than non-AF patients. Several other studies have concluded that POAF patients have a significantly longer hospital stay with a variation by region, in Asia the excess hospital stay is 4.99 days (34, 35) compared to the USA, where the excess stay is 3.2 days (36).

### Strengths and limitations

The current study analyses the association between cytokines and POAF after cardiac surgery in one of the largest series to date. However, the results of the current study should be viewed as hypothesis-generating since the study is a post-hoc analysis of the trial, and it was not specifically powered to assess the effect of cytokines on the development of POAF, and it was powered to assess the effect RIPC on cytokines as part of the original trial. Furthermore, we have attempted to construct a prediction model in a relatively small sample size; therefore, we have to acknowledge the possible risk of overfitting in our prediction analysis. More extensive studies are needed to assess the external validity of our results.

## Conclusions

Our study suggests no significant association exists between cytokine release patterns and the development of POAF in patients undergoing coronary artery bypass surgery or aortic valve surgery. Age and Aortic Cross-clamp time were found to be significant predictors of POAF.

## Data Availability

The original contributions presented in the study are included in the article/**Supplementary Material**, further inquiries can be directed to the corresponding author.

## References

[1] Zakkar M Ascione R James AF Angelini GD Suleiman MS. Inflammation, oxidative stress and postoperative atrial fibrillation in cardiac surgery. Pharmacol Ther. (2015) 154:13–20. 10.1016/j.pharmthera.2015.06.009 26116810

[2] Benedetto U Gaudino MF Dimagli A Gerry S Grey A Lees B Postoperative atrial fibrillation and long-term risk of stroke after isolated coronary artery bypass graft surgery. Circulation. (2020) 142:1320–9. 10.1161/CIRCULATIONAHA.120.046940 33017213 PMC7845484

[3] Helgadottir S Sigurdsson MI Ingvarsdottir IL Arnar DO Gudbjartsson T. Atrial fibrillation following cardiac surgery: risk analysis and long-term survival. J Cardiothorac Surg. (2012) 7:87. 10.1186/1749-8090-7-87 22992266 PMC3515503

[4] Ziabakhsh-Tabari S. Can perioperative C-reactive protein and interleukin-6 levels predict atrial fibrillation after coronary artery bypass surgery? Saudi Med J. (2008) 29:1429–31. 18946567

[5] Zheng Y Liu Y Liu Y. The relationship between inflammatory biomarkers and postoperative atrial fibrillation after cardiac surgery: a systematic review and meta-analysis. Chin J Clin Thorac Cardiovasc Surg. (2021) 28:122–4. https://pesquisa.bvsalud.org/portal/resource/pt/wpr-881224?lang=en (Accessed January 16, 2023).

[6] Mohamed AA Nor El-Dien DM. Preoperative serum levels of interleukin-6 and interleukin-8 as predictors of the development of postoperative atrial fibrillation among patients undergoing coronary artery bypass grafting surgery. J Cardiothorac Vasc Anesth. (2013) 7:50. 10.4103/1687-9090.124029

[7] Wu Z-K Laurikka J Vikman S Nieminen R Moilanen E Tarkka MR. High postoperative interleukin-8 levels related to atrial fibrillation in patients undergoing coronary artery bypass surgery. World J Surg. (2008) 32:2643–9. 10.1007/s00268-008-9758-7 18850246

[8] Zhou X Dudley SC. Evidence for inflammation as a driver of atrial fibrillation. Front Cardiovasc Med. (2020) 7:62. 10.3389/fcvm.2020.00062 32411723 PMC7201086

[9] Moscarelli M Fiorentino F Suleiman M-S Emanueli C Reeves BC Punjabi PP Remote ischaemic preconditioning in isolated aortic valve and coronary artery bypass surgery: a randomized trial†. Eur J Cardiothorac Surg. (2018) 55:905–12. 10.1093/ejcts/ezy404 PMC6477640 30544237

[10] Motulsky HJ Brown RE. Detecting outliers when fitting data with nonlinear regression—a new method based on robust nonlinear regression and the false discovery rate. BMC Bioinformatics. (2006) 7:123. 10.1186/1471-2105-7-123 16526949 PMC1472692

[11] Curran-Everett D Milgrom H. Post-hoc data analysis. Curr Opin Allergy Clin Immunol. (2013) 13:223–4. 10.1097/ACI.0b013e3283609831 23571411

[12] Srinivas TR Ho B Kang J Kaplan B. Post hoc analyses. Transplantation. (2015) 99:17–20. 10.1097/TP.0000000000000581 25525920

[13] Warltier DC Laffey JG Boylan JF Cheng DCH. The systemic inflammatory response to cardiac surgery. Anesthesiology. (2002) 97:215–52. 10.1097/00000542-200207000-00030 12131125

[14] Muckart DJ Bhagwanjee S. American college of chest physicians/society of critical care medicine consensus conference definitions of the systemic inflammatory response syndrome and allied disorders in relation to critically injured patients. Crit Care Med. (1997) 25:1789–95. 10.1097/00003246-199711000-00014 9366759

[15] Träger K Fritzler D Fischer G Schröder J Skrabal C Leibold A. Treatment of post-cardiopulmonary bypass sirs by hemoadsorption: a case series. Int J Artif Organs. (2016) 39:141–6. 10.5301/ijao.5000492 27140295

[16] Day JRS Taylor KM. The systemic inflammatory response syndrome and cardiopulmonary bypass. Int J Surg. (2005) 3:129–40. 10.1016/j.ijsu.2005.04.002 17462274

[17] Roach GW Kanchuger M Mangano CM Newman M Nussmeier N Wolman R Adverse cerebral outcomes after coronary bypass surgery. N Engl J Med. (1996) 335:1857–64. 10.1056/NEJM199612193352501 8948560

[18] Mangano DT. Effects of acadesine on myocardial infarction, stroke, and death following surgery: a meta-analysis of the 5 international randomized trials. JAMA. (1997) 277:325. 10.1001/jama.1997.03540280063035 9002496

[19] Christenson J. Adult respiratory distress syndrome after cardiac surgery. Cardiovasc Surg. (1996) 4:15–21. 10.1016/0967-2109(96)83778-1 8634840

[20] Kawamura T Wakusawa R Okada K Inada S. Elevation of cytokines during open heart surgery with cardiopulmonary bypass: participation of interleukin 8 and 6 in reperfusion injury. Can J Anaesth. (1993) 40:1016–21. 10.1007/BF03009470 8269560

[21] McBride WT Armstrong MA Crockard AD McMurray TJ Rea JM. Cytokine balance and immunosuppressive changes at cardiac surgery: contrasting response between patients and isolated CPB circuits. Br J Anaesth. (1995) 75:724–33. 10.1093/bja/75.6.724 8672321

[22] Sablotzki A Mann V Simm A Czeslick E. Veränderungen des Zytokin-Netzwerkes bei eskalierendem sirs nach Herzchirurgischen Operationen [Changes in the cytokine network through escalating SIRS after heart surgery]. Anasthesiol Intensivmed Notfallmed Schmerzther. (2001) 36:552–9. 10.1055/s-2001-17262 11577354

[23] Ucar H Tok M Atalar E Dogan OF Mehmet O Farsak B Predictive significance of plasma levels of interleukin-6 and high-sensitivity C-reactive protein in atrial fibrillation after coronary artery bypass surgery. Heart Surg Forum. (2007) 10:131–5. 10.1532/hsf98.20061175 17597037

[24] Elahi MM Flatman S Matata BM. Tracing the origins of postoperative atrial fibrillation: the concept of oxidative stress-mediated myocardial injury phenomenon. Eur J Cardiovasc Prev Rehabil. (2008) 15:735–41. 10.1097/HJR.0b013e328317f38a 19020458

[25] Wu N Xu B Xiang Y Wu L Zhang Y Ma X Association of inflammatory factors with occurrence and recurrence of atrial fibrillation: a meta-analysis. Int J Cardiol. (2013) 169:62–72. 10.1016/j.ijcard.2013.08.078 24095158

[26] Maesen B Nijs J Maessen J Allessie M Schotten U. Post-operative atrial fibrillation: a maze of mechanisms. Europace. (2012) 14:159–74. 10.1093/europace/eur208 21821851 PMC3262403

[27] Ishida K Kimura F Imamaki M Ishida A Shimura H Kohno H Relation of inflammatory cytokines to atrial fibrillation after off-pump coronary artery bypass grafting. Eur J Cardiothorac Surg. (2006) 29:501–5. 10.1016/j.ejcts.2005.12.028 16439145

[28] Mathew JP. A multicenter risk index for atrial fibrillation after cardiac surgery. JAMA. (2004) 291:1720. 10.1001/jama.291.14.1720 15082699

[29] Todorov H Janssen I Honndorf S Bause D Gottschalk A Baasner S Clinical significance and risk factors for new onset and recurring atrial fibrillation following cardiac surgery—a retrospective data analysis. BMC Anesthesiol. (2017) 17:163. 10.1186/s12871-017-0455-7 29197340 PMC5712135

[30] Turkkolu ST Selçuk E Köksal C. Biochemical predictors of postoperative atrial fibrillation following cardiac surgery. BMC Cardiovasc Disord. (2021) 21:167. 10.1186/s12872-021-01981-z 33836659 PMC8033715

[31] Hashemzadeh K Dehdilani M Dehdilani M. Postoperative atrial fibrillation following open cardiac surgery: predisposing factors and complications. J Cardiovasc Thorac Res. (2013) 5:101–7. 10.5681/jcvtr.2013.022 24252985 PMC3825396

[32] Qureshi M Ahmed A Massie V Marshall E Harky A. Determinants of atrial fibrillation after cardiac surgery. Rev Cardiovasc Med. (2021) 22:329. 10.31083/j.rcm2202040 34258901

[33] Park TJ Shah D Gillard P Ferguson WG Subacius H Gray E Abstract 9901: in-hospital and 30-day post-discharge outcomes associated with postoperative atrial fibrillation: a contemporary analysis from the society of thoracic surgeons adult cardiac surgery database. Circulation. (2022):146.

[34] Ghurram A Krishna N Bhaskaran R Kumaraswamy N Jayant A Varma PK. Patients who develop post-operative atrial fibrillation have reduced survival after off-pump coronary artery bypass grafting. Indian J Thorac Cardiovasc Surg. (2019) 36:6–13. 10.1007/s12055-019-00844-9 32435088 PMC7222924

[35] Pardo D Shroyer AL Bilfinger TV. Global variation in the incidence of new-onset postoperative atrial fibrillation after cardiac and non-cardiac surgery: a systematic review. Vessel Plus. (2022) 6:50. 10.20517/2574-1209.2021.146

[36] Tamis JE Steinberg JS. Atrial fibrillation independently prolongs hospital stay after coronary artery bypass surgery. Clin Cardiol. (2000) 23:155–9. 10.1002/clc.4960230305 10761801 PMC6654937

