# Corrigendum: Patterns of cytokine release and association with new onset of post-cardiac surgery atrial fibrillation

**DOI:** 10.3389/fsurg.2024.1320769

**Published:** 2024-01-11

**Authors:** Rahul Kota, Marco Gemelli, Arnaldo Dimagli, M.-Saadeh Suleiman, Marco Moscarelli, Tim Dong, Gianni Angelini, Daniel P. Fudulu

**Affiliations:** ^1^ Bristol Medical School, Faculty of Health Sciences, University of Bristol, Bristol, United Kingdom; ^2^ Department of Cardiac Surgery, Bristol Heart Institute, Bristol, United Kingdom; ^3^ University of Bristol, Bristol, United Kingdom

A Corrigendum on Patterns of cytokine release and association with new onset of post-cardiac surgery atrial fibrillation By Kota R, Gemelli M-S, Dimagli A, Suleiman S, Moscarelli M, Dong T, Angelini G, Fudulu DP. (2023). Front Surg. 10:1205396. doi: 10.3389/fsurg.2023.1205396

Incorrect Funding

In the published article, there was an error in the Funding statement. The authors did not include BHF grant number, and mistakenly declared the research was not funded. The original article states ‘No funding was received for the research, authorship, and/or publication of this article’. The correct Funding statement appears below.

