# Supplementary material for: Patterns of cytokine release and association with new onset of post-cardiac surgery atrial fibrillation
Source: Front Surg. 2023 May 30;10:1205396. doi: 10.3389/fsurg.2023.1205396 (PMC10266410; doi:10.3389/fsurg.2023.1205396)
Supplement: Supplementary file 1 [file Table1.docx]

Supplementary material

Title Page

Official Title: Patterns of cytokine release and association with new onset of post-cardiac surgery atrial fibrillation

Table 1 The effect of IL-6, IL-0, IL-8 and TNFα in logistic regression models for prediction of POAF in patients undergoing AVR.

|  | **IL-6 model** | | | **IL-10 model** | | | **IL-8 model** | | | **TNF**α**-model** | | |
| --- | --- | --- | --- | --- | --- | --- | --- | --- | --- | --- | --- | --- |
| *Predictors* | *Odds Ratios* | *CI* | *p* | *Odds Ratios* | *CI* | *p* | *Odds Ratios* | *CI* | *p* | *Odds Ratios* | *CI* | *p* |
| Age (years) | 1.09 | 1.03 – 1.17 | **0.006** | 1.08 | 1.03 – 1.16 | **0.009** | 1.08 | 1.03 – 1.16 | **0.009** | 1.09 | 1.03 – 1.16 | **0.007** |
| Aortic cross clamp time (min) | 1.02 | 0.99 – 1.06 | 0.181 | 1.02 | 0.99 – 1.06 | 0.255 | 1.02 | 0.99 – 1.06 | 0.255 | 1.02 | 0.99 – 1.06 | 0.162 |
| IL-6 (pg/mL) | 1.00 | 0.99 – 1.01 | 0.927 |  |  |  |  |  |  |  |  |  |
| IL-10 (pg/mL) |  |  |  | 1.03 | 0.99 – 1.07 | 0.199 |  |  |  |  |  |  |
| IL-8 ((pg/mL) |  |  |  |  |  |  | 1.03 | 0.99 – 1.07 | 0.199 |  |  |  |
| TNFα (pg/mL) |  |  |  |  |  |  |  |  |  | 1.01 | 0.98 – 1.04 | 0.552 |

Table 2 The effect of IL-6, IL-0, IL-8 and TNFα in logistic regression models for prediction of POAF in patients undergoing CABG.

|  | **IL-6 model** | | | **IL-10 model** | | | **IL-8 model** | | | **TNF**α**-model** | | |
| --- | --- | --- | --- | --- | --- | --- | --- | --- | --- | --- | --- | --- |
| *Predictors* | *Odds Ratios* | *CI* | *p* | *Odds Ratios* | *CI* | *p* | *Odds Ratios* | *CI* | *p* | *Odds Ratios* | *CI* | *p* |
| Age (years) | 1.04 | 0.95 – 1.16 | 0.392 | 1.04 | 0.95 – 1.16 | 0.418 | 1.04 | 0.95 – 1.16 | 0.418 | 1.04 | 0.95 – 1.16 | 0.410 |
| Aortic cross clamp time (min) | 1.06 | 1.00 – 1.15 | 0.075 | 1.05 | 0.99 – 1.12 | 0.126 | 1.05 | 0.99 – 1.12 | 0.126 | 1.05 | 0.99 – 1.12 | 0.126 |
| IL-6 (pg/mL) | 1.00 | 0.99 – 1.00 | 0.158 |  |  |  |  |  |  |  |  |  |
| IL-10 (pg/mL) |  |  |  | 1.00 | 0.98 – 1.01 | 0.897 |  |  |  |  |  |  |
| IL-8 ((pg/mL) |  |  |  |  |  |  | 1.00 | 0.98 – 1.01 | 0.897 |  |  |  |
| TNFα (pg/mL) |  |  |  |  |  |  |  |  |  | 0.99 | 0.93 – 1.04 | 0.834 |
